# Supplementary material for: Quantum-circuit refrigerator
Source: Nat Commun. 2017 May 8;8:15189. doi: 10.1038/ncomms15189 (PMC5424155; doi:10.1038/ncomms15189)
Supplement: Supplementary Information — Supplementary figures, supplementary tables, supplementary notes and supplementary references. [file ncomms15189-s1.pdf]

SUPPLEMENTARY INFORMATION

Supplementary Note 1 | Temperature calibration in NIS thermometry

The temperatures of the QCR and of the probe are each measured with a pair of current-biased NIS junctions. For elastic single-electron tunnelling, the current through an NIS junction with tunnelling resistance R_T is given by[1]

$$I(V,T_\mathrm{e}) = \frac{1}{eR_\mathrm{T}}\int_{-\infty}^{\infty} \mathrm{d}E\, n_\mathrm{S}(E)[f(E-eV,T_\mathrm{e}) - f(E+eV,T_\mathrm{e})] \tag{1}$$

where $T_\mathrm{e} \in \{T_\mathrm{QCR}, T_\mathrm{probe}\}$ is the electron temperature of the normal-metal resistor, and V the voltage across the NIS junction. The Fermi–Dirac distribution is given by

$$f(E,T) = \frac{1}{e^{E/(k_\mathrm{B}T)}+1} \tag{2}$$

and the quasiparticle density of states in the superconductor can be parametrized by

$$n_\mathrm{S}(E) = \left|\mathrm{Re}\frac{E/\Delta + i\gamma_\mathrm{D}}{\sqrt{(E/\Delta + i\gamma_\mathrm{D})^2 - 1}}\right| \tag{3}$$

The Dynes parameter γ_D accounts, for example, for deep-sub-gap ($|V| \ll \Delta/e$) leakage current. Experimentally, γ_D and Δ are obtained by fitting supplementary equation 1 to the current–voltage characteristics of the NIS junctions used for thermometry. At sub-gap voltages, supplementary equation 1 exhibits a strong dependence on the normal-metal temperature, and hence an NIS junction can be utilized as a secondary thermometer to probe the electron temperature of the normal metal. In our experiments, we apply constant current bias, $I_\mathrm{th,QCR/probe}$, across the thermometer junctions and measure the voltage drop across each pair to obtain the signal used to extract the electron temperature.

At high bath temperatures, the electron temperature of the normal metal follows the bath temperature, T_0, giving rise to a faithful conversion function, g, of the observed thermometer voltage, $V(T_0) = g^{-1}(T_0)$, into electron temperature, $T_\mathrm{e} = g(V)$. At low temperatures however, the electrons thermally decouple from the phonons leading to a saturation of the electron temperature with decreasing phonon temperature. Before the saturation, the thermometer voltage depends rather linearly on the bath temperature as shown in Supplementary Fig. 1a. Throughout this paper, we employ such linear conversion from the thermometer voltage to the electron temperature independently for each thermometer.

Supplementary Note 2 | Thermal Model

Our thermal model is presented in Fig. 3c. Several heat transport mechanisms are responsible for the observed temperature of the probe resistor: Firstly, the NIS junctions in the QCR lead to exchange of energy with the resonator due to photon-assisted tunnelling, P_T. Secondly, the heat exchange between the normal-metal electrons and the resonator is governed by ohmic losses, $P_{\Gamma,\mathrm{probe/QCR}}$. Thirdly, the normal-metal electrons are coupled to the phonon bath leading to the power flow P_ep. Fourthly, our model accounts for weak residual heating of the probe due to the power dissipation at the QCR, P_res, and leakage of photons to the resonator from high-temperature stages of the cryostat, P_leak. Finally, we include an excess power P_x due to a constant thermal conductance G_x to an excess reservoir at temperature T_x. Supplementary Table 1 shows the values of the parameters used in the model.

In our thermal model (Fig. 3c), the electron temperature of the probe resistor for a given QCR temperature and operation voltage may be solved from the power balance equation

$$P_{\Gamma,\mathrm{probe}} - P_\mathrm{ep} - P_\mathrm{res} - P_\mathrm{x} = 0 \tag{4}$$

The power flowing from the probe electrons to the resonator photons due to ohmic losses can be expressed as[2]

$$P_{\Gamma,\mathrm{probe}} = \Gamma^\mathrm{probe}_{0\to1} p_0 \hbar\omega_0 - \Gamma^\mathrm{probe}_{1\to0} p_1 \hbar\omega_0 \tag{5}$$

where $\Gamma^\mathrm{probe}_{0\to1}$ and $\Gamma^\mathrm{probe}_{1\to0}$ are the excitation and relaxation rates of the resonator photons due to the probe resistor, respectively, and $p_0 = 1 - p_1$ is the probability of the resonator to be in its quantum-mechanical ground state. For simplicity, we consider here only the two lowest-energy states of the resonator. In the steady state achieved in our experiments, we have

$$\dot{p}_0 = 0 = -\Gamma^+ p_0 + \Gamma^- p_1 \quad \Rightarrow \quad p_0 = \frac{\Gamma^-}{\Gamma^- + \Gamma^+}, \quad p_1 = \frac{\Gamma^+}{\Gamma^- + \Gamma^+} \tag{6}$$

where the total excitation and relaxation rates of the resonator mode are given by

$$\begin{aligned} \Gamma^+ &= \Gamma^\mathrm{QCR}_{0\to1} + \Gamma^\mathrm{probe}_{0\to1} + \Gamma^\mathrm{T}_{0\to1} + \Gamma_\mathrm{leak} \\ \Gamma^- &= \Gamma^\mathrm{QCR}_{1\to0} + \Gamma^\mathrm{probe}_{1\to0} + \Gamma^\mathrm{T}_{1\to0} \end{aligned} \tag{7}$$

respectively. Here, the rate Γ_leak determines the leakage power to the resonator $P_\mathrm{leak} = \hbar\omega_0\Gamma_\mathrm{leak}p_0$ and the rate Γ^T arises from the photon-assisted tunnelling at the QCR as described in the next section. The rates arising from the ohmic losses are given by[2]

$$\Gamma^\mathrm{QCR/probe}_{0\to1} = \frac{\gamma}{e^{\hbar\omega_0/(k_\mathrm{B}T_\mathrm{QCR/probe})} - 1}, \quad \Gamma^\mathrm{QCR/probe}_{1\to0} = \frac{\gamma}{1 - e^{-\hbar\omega_0/(k_\mathrm{B}T_\mathrm{QCR/probe})}} \tag{8}$$

where we employ the same base rate $\gamma = 2\omega_0 R \sin^2(\pi x/L)/(\pi Z_0)$ for the QCR and the probe due to the symmetry in their resistances R and distances from the edge of the resonator x. The characteristic impedance $Z_0 = \sqrt{L_l/C_l}$ of the resonator of length L is given by the inductance and capacitance per unit length L_l and C_l, respectively.

supplementary equation 4 also includes the term P_{ep} that arises from the coupling between the normal-metal electrons and the substrate phonons. This heat flow is given by[1]

$$P_{ep} = \Sigma_{Cu}\Omega_{probe}(T_0^5 - T_{probe}^5) \tag{9}$$

where Ω_{probe} is the volume of the probe resistor and $\Sigma_{Cu} = 2 \times 10^9$ W K^{-5} m^{-3} is the known electron–phonon coupling constant of copper[1].

In the control sample, we observed weak heating of the probe due to the power dissipation at the QCR. This heating is approximately linearly dependent on the bath temperature, and hence we include a residual heating power to our thermal model in the form

$$P_{res} = \alpha(\beta - T_0)I_{QCR}V_{QCR} \tag{10}$$

We fix the values of the parameters α and β (see Supplementary Table 1) using the measurement data of the control sample and use the same values also in the case of the active sample. Importantly, the contribution of this residual heating at subgap voltages is much weaker than that of the other heat conduction mechanisms. The microscopic origin of the residual heating remains to be studied further but the existence of such very weak channel is not surprising.

In supplementary equation 4, we choose the excess power to assume the form

$$P_x = G_x(T_x - T_{probe}) \tag{11}$$

where, for simplicity, G_x is a constant thermal conductance and T_x is the temperature of the excess bath. We assume that the dominating thermal coupling of the excess bath to the phonon bath is through electron–phonon coupling and that the excess bath is so large that its temperature is essentially independent of the temperature of the probe. However, we assume a constant heating power, P_x^{con}, at the excess bath which leads to a finite saturation temperature, T_x^{sat}, even at zero phonon bath temperature. Equating P_x^{con} with the power due to the electron–phonon coupling (see supplementary equation 9) yields $T_x = [(T_x^{sat})^5 + T_0^5]^{1/5}$.

We adjust G_x and T_x^{sat} to match T_{probe} predicted by the thermal model to that measured at bath temperatures $T_0 = 25$ mK and 50 mK without operating the QCR ($V_{QCR} = 0$).

Supplementary Fig. 1b shows the measured probe temperature in this case together with the prediction of the thermal model. Although the model is fitted to the measured data only at the two lowest bath temperatures, very good agreement with the experimental results and the theoretical prediction is achieved in the whole temperature regime, in which the linear temperature calibration is valid.

As described below, there are no free parameters in the resonator excitation and relaxation rates that give rise to the power

$$P_{\mathrm{T}} = \hbar\omega_0(\Gamma^{\mathrm{T}}_{0\to1}p_0 - \Gamma^{\mathrm{T}}_{1\to0}p_1) \tag{12}$$

from the photon-assisted tunnelling. Thus Γ_{leak} is the only parameter we adjust to fit the thermal model to the temperature drops observed in Figs. 3 and 4 at the probe due to the QCR. Since we adjust the value of Γ_{leak} to obtain a good match at the lowest bath temperature $T_0 = 25$ mK, the results of the thermal model at higher temperatures such as those at $T_0 = 150$ mK in Fig. 3a may be considered as a theoretical prediction.

Supplementary Note 3 | Photon-assisted single-electron tunnelling

In our case, the single-electron tunnelling through the NIS junctions can be described by Fermi golden rule taking into account the voltage fluctuations arising from the electromagnetic environment of the junction. In this $P(E)$ theory[3], the forward tunnelling rate, i.e., the rate for an electron to tunnel from the normal metal to the superconductor, is given by[3,4]

$$\vec{\Gamma}(V_{\mathrm{QCR}}) = \frac{1}{e^2R_{\mathrm{T}}}\int_{-\infty}^{\infty}\int_{-\infty}^{\infty}\mathrm{d}E\,\mathrm{d}E'\,n_{\mathrm{S}}(E')f_{\mathrm{N}}(E-eV_{\mathrm{QCR}})[1-f_{\mathrm{S}}(E')]P(E-E') \tag{13}$$

where the Fermi distribution functions $f_{\mathrm{N}}(E) = f(E, T_{\mathrm{QCR}})$ and $f_{\mathrm{S}}(E) = f(E, T_0)$ are given by supplementary equation 2 and $P(E)$ is the probability density function for the environment to absorb E amount of energy. For simplicity, we have assumed above that the quasiparticle excitations in the superconductor are well thermalized with the phonon bath.

In the zero-temperature limit for the fundamental mode of the resonator acting as the environment, we have for an NIS tunnel junction[3]

$$P(E) \approx e^{-\rho}\sum_{k=0}^{\infty}\frac{\rho^k}{k!}\delta(E-k\hbar\omega_0) = \sum_{k=0}^{\infty}q_k\delta(E-k\hbar\omega_0), \tag{14}$$

where ρ is an environmental parameter

$$\rho = \frac{\pi}{CR_{\mathrm{K}}\omega_0} \tag{15}$$

that depends on the effective capacitance, $C = |L/2 - x|C_l/2 \approx LC_l/2$ for $x \ll 1$, of the LC oscillator which is used to model the fundamental mode. Here, $R_K = 25.8$ kΩ is the von Klitzing constant. The coefficient q_k equals the probability of emitting k quanta of energy to the resonator in the course of single-electron tunnelling. Since $\rho = 4.7 \times 10^{-3} \ll 1$ with our parameters (see Supplementary Table 1), the elastic tunnelling events, $k = 0$, for which no heat exchange with the resonator takes place, clearly dominate in the probabilities.

In our case of finite temperature, the probability for the tunnelling electron to absorb a quantum of energy from the resonator, q_{-1}, is related to the emission probability by the detailed-balance condition $q_{-1} = q_1 \exp[-\hbar\omega_0/(k_B T_r)]$, where T_r is the temperature of the resonator. For simplicity, we consider only zero- and single-photon events, justified by $\rho \ll 1$.

In the following, we consistently assume the low-temperature limit for the resonator, which is required by the fact that we take only the zero- and single-photon states of the lowest resonator mode into account. Thus we obtain the approximate probabilities

$$q_0 = \frac{1}{1+\rho}, \quad q_1 = \frac{\rho}{1+\rho} \times \frac{1}{1+e^{-\hbar\omega_0/(k_B T_r)}}, \quad q_{-1} = \frac{\rho}{1+\rho} \times \frac{e^{-\hbar\omega_0/(k_B T_r)}}{1+e^{-\hbar\omega_0/(k_B T_r)}} \tag{16}$$

and

$$P(E) = \sum_{k=-1}^{1} q_k \delta(E - k\hbar\omega_0) \tag{17}$$

Using supplementary equations 13, 16 and 17, we obtain the tunnelling rates for the electrons in the forward direction, $\vec{\Gamma}$. The backward rate $\overleftarrow{\Gamma}$ can be obtained in a similar fashion[3]. The rates can further be expressed as sums of contributions from the different processes: emission ($\vec{\Gamma}_1$ and $\overleftarrow{\Gamma}_1$), absorption ($\vec{\Gamma}_{-1}$ and $\overleftarrow{\Gamma}_{-1}$), and elastic tunnelling ($\vec{\Gamma}_0$ and $\overleftarrow{\Gamma}_0$). These electron tunnelling rates are distinct from the photon-assisted resonator excitation and relaxation rates in supplementary equation 7 which can be expressed as

$$\begin{aligned} \Gamma^{T}_{0\to1} &= \vec{\Gamma}^{T}_{0\to1} + \overleftarrow{\Gamma}^{T}_{0\to1} \\ \Gamma^{T}_{1\to0} &= \vec{\Gamma}^{T}_{1\to0} + \overleftarrow{\Gamma}^{T}_{1\to0} \end{aligned} \tag{18}$$

where $\vec{\Gamma}^{T}_{0\to1} = \vec{\Gamma}_1/p_0$, $\overleftarrow{\Gamma}^{T}_{0\to1} = \overleftarrow{\Gamma}_1/p_0$, $\vec{\Gamma}^{T}_{1\to0} = \vec{\Gamma}_{-1}/p_1$, and $\overleftarrow{\Gamma}^{T}_{1\to0} = \overleftarrow{\Gamma}_{-1}/p_1$ since the average number of electrons tunnelled in a given process is equal to the average number of photons exchanged in this process. Here, the direct dependence of the resonator excitation and relaxation rates on the temperature of the resonator is canceled by the temperature dependence of the resonator populations (see supplementary equation 6). Thus the resonator experiences the

QCR as a voltage-tunable environment. Using the above results, the forward resonator rates assume the forms

$$\begin{aligned}\vec{\Gamma}^{\mathrm{T}}_{0\to1} &= \frac{\rho}{1+\rho}\frac{1}{e^2R_{\mathrm{T}}}\int_{-\infty}^{\infty}\mathrm{d}E\, f_{\mathrm{N}}(E-eV)n_{\mathrm{S}}(E-\hbar\omega_0)[1-f_{\mathrm{S}}(E-\hbar\omega_0)]\\ \vec{\Gamma}^{\mathrm{T}}_{1\to0} &= \frac{\rho}{1+\rho}\frac{1}{e^2R_{\mathrm{T}}}\int_{-\infty}^{\infty}\mathrm{d}E\, f_{\mathrm{N}}(E-eV)n_{\mathrm{S}}(E+\hbar\omega_0)[1-f_{\mathrm{S}}(E+\hbar\omega_0)]\end{aligned} \tag{19}$$

Thus the photon-assisted resonator relaxation and excitation rates (supplementary equation 18) can be theoretically predicted without any free parameters. Consequently, these rates affect the power flowing into the probe resistor through supplementary equations 5–7.

Supplementary Note 4 | Minimizing undesired losses due to the QCR

Internal quality factors of a bare superconducting coplanar-waveguide resonators, $Q_{\mathrm{int,bare}}$, of the order $\sim 10^6$ have been demonstrated in the single-photon regime[6–8]. Such state-of-the-art values may be obtained with sophisticated fabrication techniques employing proper choices of materials such as TiN on a high-purity silicon substrate[7,9,10]. In this section, we discuss the sources of dissipation added by the introduction of the QCR into the resonator and give a sample design which is optimized for low losses although not hindering the desired operation characteristics of the QCR. Using realistically achievable parameters, our analysis indicates that the optimized design allows us to make the additional losses due to the QCR small compared with an internal quality factor of 10^6. Importantly, the optimized design is also compatible with the fabrication techniques of the low-loss resonators and other superconducting quantum devices, and hence the QCR holds great potential in introducing temporally controlled dissipation without degrading the coherence properties when inactive.

In this paper, we measure the resonator temperature using a probe resistor that couples through ohmic losses to the resonator. However, such dissipative measurement technique is not necessary in the future. For example, if the QCR is used to cool a high-quality resonator, the photon occupation numbers may be measured using a dispersively coupled superconducting qubit[11]. Thus we consider below a case, in which there is no probe resistor in the system. In this case, we differentiate three possible sources of dissipation: ohmic losses at the QCR, losses due to the smearing of the superconductor density of states, and losses at the metal insulator interfaces. We discuss each of these below. In addition, we investigate in the next section the losses owing to the photon-assisted tunnelling giving rise to the operation of the QCR. Classically, the normal-metal resistor, R, of the QCR introduces

dissipation in the resonator mode due to the electric current, $I(x)$, carried by the excitations of the mode and the Ohm's law $P_{\rm res} = RI^2(x)$. Thus it is natural that the ohmic losses can be greatly reduced by reducing the resistance value and moving the resistor close to the end of the resonator where the current profile of the mode linearly vanishes. Employing the quantum-mechanical treatment used in supplementary equation 8, the internal quality factor due to this loss mechanism only assumes the form[2]

$$Q_{\rm int,ohm} \approx \frac{\omega_0}{\gamma} \tag{20}$$

where $\gamma = 2\omega_0 R\sin^2(\pi x/L)/(\pi Z_0)$ is the resonator internal dissipation rate due to ohmic losses. For the optimized sample design shown in Supplementary Fig. 2a, the resistor is at the very end of the resonator, and hence corrections to the mode current profile from the total junction capacitance, $C_{\rm J}^{\rm tot}$, are significant. Thus we estimate the effective distance from the resonator end to be given by $x = C_{\rm J}^{\rm tot}/C_{\rm l} = 3.4\ \mu$m. Together with the resistance $R = 0.3\ \Omega$ of the copper block this implies $Q_{\rm int,ohm} = 1.3 \times 10^8$. Another way to arrive at an equal $Q_{\rm int,ohm}$ is the following: (i) treat the resistor and the junction capacitors as a lumped-element termination impedance, Z, for the resonator, (ii) calculate the current through the impedance, $I_{\rm z}$, using the impedance Z and the undisturbed voltage of the resonator mode, and (iii) obtain the dissipated power from Ohm's law, $RI_{\rm z}^2$. Hence these losses have a negligible effect on the total internal quality factor of the resonator assuming that $Q_{\rm int,bare} = 10^6$.

The Dynes density of states for the tunnel junctions of the QCR may also contribute to the internal loss of the resonator. For a single NIS junction, we estimate this loss as

$$Q_{\rm int,Dynes} = 2\pi \times \frac{n\hbar\omega_0}{P_{\rm Dynes}/f_0} = \frac{n\hbar\omega_0^2}{P_{\rm Dynes}} \tag{21}$$

where $P_{\rm Dynes} = (\langle \hat{V}_{\rm res}\rangle^2 - \text{z.p.f})/R_{\rm Dynes}$ is the photon power dissipation due to the subgap resistance $R_{\rm Dynes} = R_{\rm T}/\gamma_{\rm D}$, n is the average photon number, and z.p.f. denotes the contribution arising from the zero-point voltage fluctuations. The voltage operator of the resonator fundamental mode $\hat{V}_{\rm res}$ is given by

$$\hat{V}_{\rm res} = \sqrt{\frac{\hbar\omega_0}{LC_{\rm l}}}(\hat{a} + \hat{a}^\dagger)\cos(\frac{\pi x}{L}) \tag{22}$$

where $\hat{a}$ and $\hat{a}^\dagger$ represent the bosonic annihilation and creation operators of the mode, respectively. Thus we may express supplementary equation 21 as

$$Q_{\mathrm{int,Dynes}} = \frac{R_{\mathrm{T}} L C_{\mathrm{l}} \omega_0}{2\gamma_{\mathrm{D}}} \cos^{-2}\left(\frac{\pi x}{L}\right) \approx \frac{\pi R_{\mathrm{T}}}{2\gamma_{\mathrm{D}} Z_0} \tag{23}$$

where the cosine term is approximated to be unity for $x \ll 1$. Even for the sample realized in this paper, supplementary equation 23 yields a Dynes quality factor well above 10^6. Thus these losses are negligible for the optimized sample, the parameters of which yield a Dynes quality factor above 10^{10} because of the larger tunnelling resistance and smaller Dynes parameter. A typical reason for the smearing of the density of states is photon-assisted tunnelling arising from noise coupled through the dc leads of the NIS junctions, which can be suppressed by introducing shunt capacitors to ground[4]. This additional photon-assisted tunnelling may be treated independent of that due to the resonator photons since the voltage fluctuations related to these two mechanisms are uncorrelated.

In addition to the above-discussed loss mechanisms, in principle, there may be additional dissipation arising from the normal-metal–insulator interfaces which have not yet been thoroughly investigated in the context of cQED. Typically, such losses are attributed to quantum fluctuators coupling to the voltage drop across the interface. Since we expect such loss mechanisms to be very weak, we have utilized in the optimized design capacitive coupling of the normal-metal to the resonator. Instead of the parallel-plate design, a finger capacitor may be used as well. Furthermore, the normal metal may as well be galvanically connected to the superconducting resonator implying essentially no voltage drop at the arising normal-metal–superconductor interface due to the large series impedance of the NIS tunnel junction, and hence no losses arise from possible fluctuators here. Although the capacitor at the NIS junction cannot be removed, the junction can be fabricated purely from the same type of aluminum as is used in the Josephson junctions of typical cQED architectures by employing the inverse proximity effect as described in ref. 12. Thus it is possible to distinguish the any unwanted dissipation arising from normal-metal–insulator interfaces.

In summary, the optimized sample design shown in Supplementary Fig. 2 is expected to add insignificant amount of undesired dissipation to the resonator mode. Since a resonator is a central component in cQED and many of the state-of-the-art qubits can be described as slightly anharmonic oscillators, these estimates suggests that the QCR can be used in the future to directly cool a multitude of quantum technological components.

Supplementary Note 5 | Temporal control of dissipation using the QCR

The QCR appears to the coupled quantum device as a dissipative environment, the temperature and the coupling strength of which can be temporally controlled using the operation voltage. Supplementary Fig. 3a shows the excitation ($\Gamma^{\mathrm{T}}_{0\to1}$) and relaxation ($\Gamma^{\mathrm{T}}_{1\to0}$) rates of the resonator mode due to the photon-assisted tunnelling at the QCR. Even for the parameters of the measured sample, this photon-assisted tunnelling rate gives rise to very weak dissipation at vanishing operation voltage in comparison to a bare internal quality factor of 10^6 (see Supplementary Fig. 3b). However, the rates increase exponentially with the operation voltage providing the possibility of a fast refrigeration of the resonator mode when desired. We find in Supplementary Fig. 3a an optimal operation voltage with respect to the temperature corresponding to the photon-assisted tunnelling. This optimal voltage depends on the Dynes parameter and the electron temperature of the normal metal. After one reaches the desired temperature of the refrigerated quantum device, it is beneficial to quickly ramp down the QCR operation voltage to zero.

Supplementary Note 6 | Temperature of the resonator during refrigeration

At high operation voltages, the QCR is not cooling the resonator mode but substantially heating it. In this operation regime, we expect to have considerable multi-photon occupation in the mode, which is not accurately captured by the two-state approximation employed above. Thus for an improved accuracy in the estimation of the resonator temperature and of the average photon number, we utilize an upgraded thermal model which includes also the multi-photon states.

In this upgraded model, supplementary equations 5–8 and 12 are replaced by the corresponding equations containing contributions from all photon number states as detailed in ref. 13. By invoking the typical assumption used in $P(E)$ theory that the resonator is in a thermal state, we may express the power flows into and out of the resonator using its temperature, or equivalently the average photon number

$$n = \frac{1}{\exp[\hbar\omega_0/(k_{\mathrm{B}}T_{\mathrm{res}})] - 1} \tag{24}$$

In the simulation, the average photon number changes according to

$$\hbar\omega_0\delta_{\mathrm{t}}n = P_{\mathrm{T}} + P_{\Gamma,\mathrm{QCR}} + P_{\Gamma,\mathrm{probe}} + P_{\mathrm{leak}} \tag{25}$$

until a stationary state is achieved. In the stationary state, the power flows (see Fig. 3c) balance each other.

Figure 3d and Supplementary Fig. 4 show the results of the upgraded thermal model: temperature and average photon number of the fundamental mode of the resonator as functions of the QCR operation voltage. At maximum cooling, the average photon number is reduced down to $n = 0.3$ using parameters corresponding to the measured sample. This number is well above the state of the art in cQED. However, the aim of this work is not to show record-low photon numbers but to introduce a quantum-circuit refrigerator. In fact, a relatively high initial photon number is beneficial in our experiments since is renders the temperature drop of the probe resistor observable when the QCR is operated. Nevertheless, the QCR is theoretically expected operate also in a state-of-the-art setup with very small photon leakage rates and very low temperatures.

Although Supplementary Fig. 4 show that the two-level approximation for the resonator mode is compromised at certain operation regimes of the QCR, this simple model captures the essential physics well and provides a quantitative match with the experimental results with as many fitting parameters as the upgraded model. Furthermore, we verified that the upgraded many-state model also yields a good quantitative match with the experiments leading to the same conclusions as the original thermal model. Thus for simplicity, we choose to work within the two-state model except for Fig. 3d and Supplementary Fig. 4.

Supplementary Note 7 | Analytical considerations of the QCR cooling power

The cooling power does not well characterize the QCR. Instead, one should consider the transition rates that the QCR induces on the quantum device. Nevertheless, let us derive analytical results for the cooling power of the QCR and compare them with the cooling power of typical NIS tunnel junctions. Note that the QCR cools the resonator mode, whereas a typical NIS junction cools the electron gas in the normal-metal part. Thus these two powers behave distinctly different as functions of the electron temperature.

Using the $P(E)$ theory and and considering only the two lowest levels of the fundamental resonator mode, the photon absorption power due to forward tunnelling across a QCR tunnel junction is given by

$$\vec{P}_{-1} = -\frac{\hbar\omega_0}{e^2 R_{\rm T}} q_{-1} \int_{-\infty}^{\infty} dE n_{\rm S}(E) f(E, T_{\rm S})[1 - f(E + \hbar\omega_0 + eV_{\rm QCR}/2, T_{\rm N})] \tag{26}$$

The corresponding photon emission power is given by

$$\vec{P}_1 = \frac{\hbar\omega_0}{e^2 R_\mathrm{T}} q_1 \int_{-\infty}^{\infty} dE n_\mathrm{S}(E) f(E, T_\mathrm{S})[1 - f(E - \hbar\omega_0 + eV_\mathrm{QCR}/2, T_\mathrm{N})] \tag{27}$$

If the quasiparticle temperatures of the normal metal, T_N, and of the superconductor, T_S, are suffciently low, $k_\mathrm{B}\ \max(T_\mathrm{N}, T_\mathrm{S}) \ll \min(\Delta, \hbar\omega_0)$,the Fermi distribution functions can be approximated with step functions. Here, the powers in supplementary equation 26 and supplementary equation 27 can then be simplified to

$$\begin{aligned}\vec{P}_{-1} &= -\frac{\hbar\omega_0}{e^2 R_\mathrm{T}} q_{-1} \int_{-\hbar\omega_0 - eV_\mathrm{QCR}/2}^{0} dE\ n_\mathrm{S}(E) \\ \vec{P}_1 &= \frac{\hbar\omega_0}{e^2 R_\mathrm{T}} q_1 \int_{\hbar\omega_0 - eV_\mathrm{QCR}/2}^{0} dE\ n_\mathrm{S}(E)\end{aligned} \tag{28}$$

For a suffciently small Dynes parameter, the density of states of the superconductor in supplementary equation 3 is given by

$$n_\mathrm{S}(E) = \frac{|E|}{\sqrt{E^2 - \Delta^2}} \theta(|E| - \Delta) \tag{29}$$

where $\theta(\bar{E})$ is the Heaviside step function. Thus integration of supplementary equation 28 yields

$$\begin{aligned}\vec{P}_{-1} &= -\frac{\hbar\omega_0\Delta}{e^2 R_\mathrm{T}} q_{-1} \sqrt{\left(\frac{eV_\mathrm{QCR}/2 + \hbar\omega_0}{\Delta}\right)^2 - 1}\ \ \theta(eV_\mathrm{QCR}/2 - \Delta + \hbar\omega_0) \\ \vec{P}_1 &= \frac{\hbar\omega_0\Delta}{e^2 R_\mathrm{T}} q_1 \sqrt{\left(\frac{eV_\mathrm{QCR}/2 - \hbar\omega_0}{\Delta}\right)^2 - 1}\ \ \theta(eV_\mathrm{QCR}/2 - \Delta - \hbar\omega_0)\end{aligned} \tag{30}$$

The step functions in the above equations imply that photon-assisted cooling of the resonator dominates over heating for $\Delta - \hbar\omega_0 < eV_\mathrm{QCR}/2 < \Delta + \hbar\omega_0$.

In typical NIS electronic coolers, the cooling power at the optimal bias point $V_\mathrm{QCR} \approx 2(\Delta - 0.66 \times k_\mathrm{B} T_\mathrm{N}/e)$ can be approximated by[5]

$$\dot{Q}_\mathrm{opt} \approx \frac{\Delta^2}{e^2 R_\mathrm{T}} \left[0.59 \left(\frac{k_\mathrm{B} T_\mathrm{N}}{\Delta}\right)^{3/2} - \sqrt{\frac{2\pi k_\mathrm{B} T_\mathrm{S}}{\Delta}} \times \exp\left(-\frac{\Delta}{k_\mathrm{B} T_\mathrm{S}}\right)\right] \tag{31}$$

At the considered low-temperature limit, the ratio of photonic and electronic cooling power is given by

$$\frac{\vec{P}_{-1}}{\dot{Q}_\mathrm{opt}} \approx \frac{\hbar\omega_0 q_{-1}}{\Delta^{1/2}} \left[\frac{\sqrt{(eV_\mathrm{QCR}/2 + \hbar\omega_0)^2 - \Delta^2}}{0.59\,(k_\mathrm{B} T_\mathrm{N})^{3/2}}\right] \approx 2.4 \times q_{-1} \left(\frac{\hbar\omega_0}{k_\mathrm{B} T_\mathrm{N}}\right)^{3/2} \tag{32}$$

where the last expression is obtained by setting $eV_{\rm QCR}/2 = \Delta$. We observe that this ratio increases with decreasing electron temperature, and may in principle, exceed unity. With our typical experimental parameters however, the ratio is of the order of ρ.

Supplementary Note 8 | Analytical considerations of the coefficient of performance

In addition to the cooling power, typical refrigerators are characterized by the coefficient of performance, η, which is the ratio of the net cooling power and the work done by the voltage source per unit time. For the QCR, we make an estimate

$$\eta_{\rm QCR} = \frac{P_\Gamma - P_{\Gamma,{\rm QCR}}}{V_{\rm QCR} I_{\rm QCR}} \approx \frac{P_\Gamma}{V_{\rm QCR} I_{\rm QCR}} < \frac{2\hbar\omega_0}{eV_{\rm QCR}} \approx \frac{\hbar\omega_0}{\Delta - \hbar\omega_0} \tag{33}$$

where we first neglect the weak ohmic losses and then employ the fact that the current related to photon absorption is lower than the total current. We also employed an operation voltage $eV_{\rm QCR}/2 \approx \Delta - \hbar\omega_0$ which is in the quantum-circuit refrigeration regime discussed in the main text. With our experimental parameters however, we fall well below the derived upper bound owing to relatively frequent elastic tunnelling compared with photon-assisted tunnelling. To increase the coefficient of performance, we may increase the photon energy or decrease the electron temperature and the Dynes parameter.

For a usual NIS cooler, the coefficient of performance at the optimal bias point is given by[1]

$$\eta_{\rm NIS} \approx 0.7 \times \frac{T_{\rm N}}{T_{\rm c}} \tag{34}$$

where $T_{\rm c}$ is the critical temperature of the superconductor. Thus typically $\eta_{\rm NIS}$ falls between 0.1 and 0.01. In contrast to $\eta_{\rm QCR}$, the above equation shows that $\eta_{\rm NIS}$ decreases with decreasing electron temperature[5], which again highlights the conceptual differences between the QCR and a typical NIS refrigerator.

Supplementary Note 9 | Losses due to high-order tunnelling

In the above theoretical considerations, we focus on sequential electron tunnelling since it accurately explains our experimental observations. In the future, it is important to study in detail how high-order processes such as Andreev reflection[14] affect the losses in the system. See refs. 15–19 for studies of Andreev reflection in NIS junctions. In this section,

we give worst-case estimates on the effect of the Andreev reflections on the QCR electron temperature and conclude that it seems not to pose a problem for the operation of the QCR.

Note that in our results above, we measure the electron temperature of the QCR and use this temperature as an input to the thermal model. Thus we implicitly include in the model any possible Andreev heating of the QCR electrons.

In the experimental QCR sample, the tunnel resistance per junction is about 23.4 kΩ. Although this is well less than 100 kΩ, and hence typically implying the existence of a so-called zero-bias anomaly[17], we cannot differentiate such anomaly in the experimental data and model the corresponding current voltage characteristics using a linear slope characteristic for ballistic Andreev reflection. In the subgap region, the ballistic Andreev current, I_{AR}, can be calculated as[18]

$$I_{\mathrm{AR}} = \frac{1}{8N}\frac{R_{\mathrm{K}}}{R_{\mathrm{T}}^2}V_{\mathrm{QCR}} \tag{35}$$

where the number of conduction channels $N = A/A_{\mathrm{ch}} \approx 2100$ is given by the junction area, $A \approx 0.063\ \mu\mathrm{m}^2$, and the area for a single channel[19], $A_{\mathrm{ch}} \approx 30\ \mathrm{nm}^2$. The Joule heating power due to this Andreev current is given by

$$P_{\mathrm{AR}} = I_{\mathrm{AR}}V_{\mathrm{QCR}}. \tag{36}$$

As a worst-case estimate for the Andreev heating, we employ $V_{\mathrm{QCR}} = 2\Delta/e$ and consequently obtain $P_{\mathrm{AR}} = 500$ aW. This worst-case heating power is low compared with the electron–phonon coupling and with the cooling power of the NIS junctions near the gap voltage, and hence Andreev Joule heating is not visible in Fig. 3a.

In the optimized sample, our worst-case estimate for the Andreev Joule heating is an order of magnitude lower, 50 aW. If this is the only heating power subject to the QCR electrons coupled to a 10-mK phonon bath, we obtain $T_{\mathrm{QCR}} = [T_0^5 + P_{\mathrm{A}}/(\Sigma_{\mathrm{Cu}}\Omega_{\mathrm{QCR}})]^{1/5} =$ 27 mK. This electron temperature is much lower than the lowest assumed temperature of the QCR in Supplementary Fig. 3, and hence will likely have a negligible effect on the optimized sample.

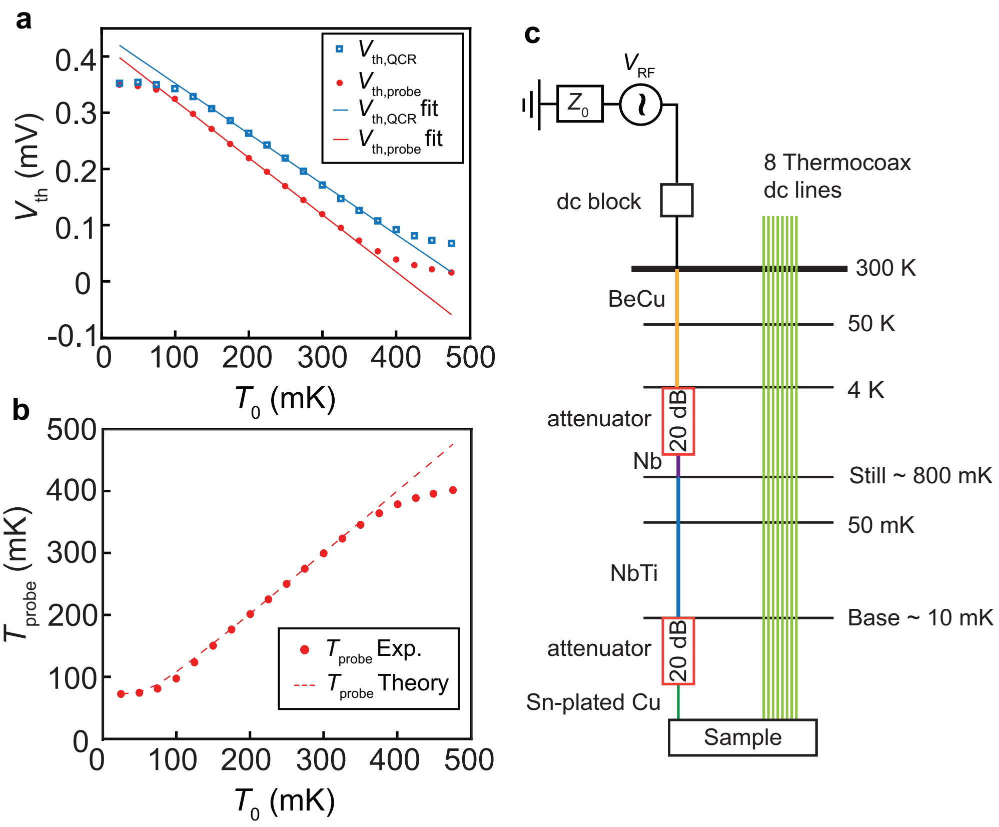

Supplementary Figure 1 | Thermometer calibration and experimental wiring. a, Thermometer voltages of the quantum-circuit refrigerator (QCR) and of the probe as functions of the phonon bath temperature. The solid lines are linear fits to the experimental data (markers) and they are used to convert the measured thermometer voltages into the electron temperatures at the QCR and at the probe. **b**, Measured electron temperature of the probe using the calibration from **a** as a function of the bath temperature. The dashed line shows the electron temperature extracted from the thermal model of Fig. 3c. The deviation of the theoretical prediction from the measurement data at high bath temperatures is due to the failure of the linear thermometer calibration also visible in **a**. In both panels, the QCR operation voltage is set to zero. **c**, Wiring scheme for the measurements. For the rf signal, 20-dB attenuators are attached at different temperature stages of the cryostat for improved thermalization. Below 4-K temperature, superconducting coaxial cables are used. Resistive Thermocoax cables are employed for the dc lines.

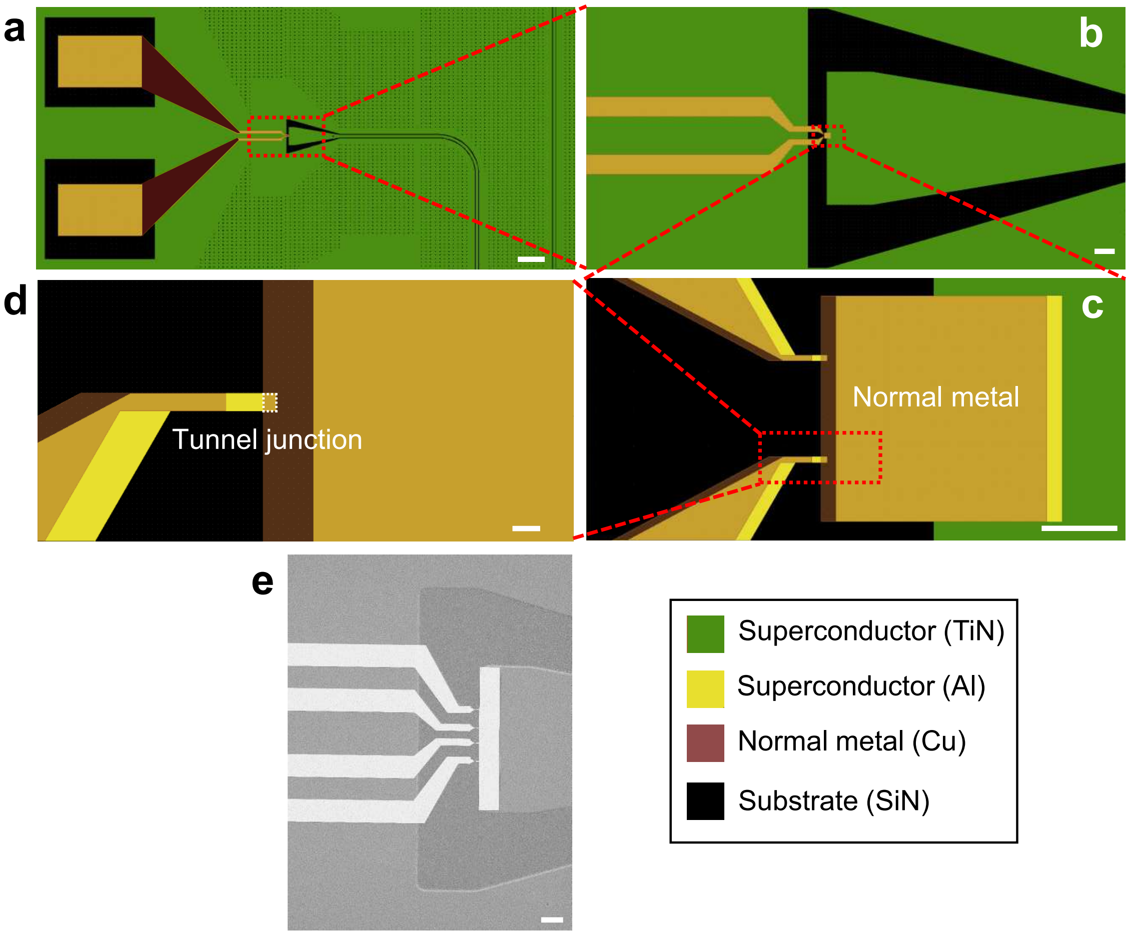

Supplementary Figure 2 | Optimized sample design. **a**, Quantum-circuit refrigerator (QCR) with two dc bias leads and bonding pads (on the left) is capacitively coupled to the end of a high-quality co-planar waveguide resonator (on the right). **b**, View of the design in the area indicated by the red rectangle in **a**. **c**, Close view of the 3×3-μm^2 copper block forming the normal metal of the QCR. The block is partially overlapping the end of the resonator centre conductor to induce capacitive coupling. Due to shadow evaporation, there is a 20-nm layer of aluminum below most of the copper parts. **d**, Close view of the bottom NIS junction (white dashed rectangle). The lithographic junction size is 50×70 nm^2, giving rise to an effective junction area of roughly 70×70 nm^2 due to the 20-nm aluminum layer. See Supplementary Table 2 for the parameters of the optimized QCR sample. **e**, SEM image of a sample similar to the optimized design but with four NIS junctions and a larger normal-metal island. The scalebars for panels **a**, **b**, **c**, **d**, and **e** correspond to 100 μm, 10 μm, 1 μm, 100 nm, and 10 μm, respectively.

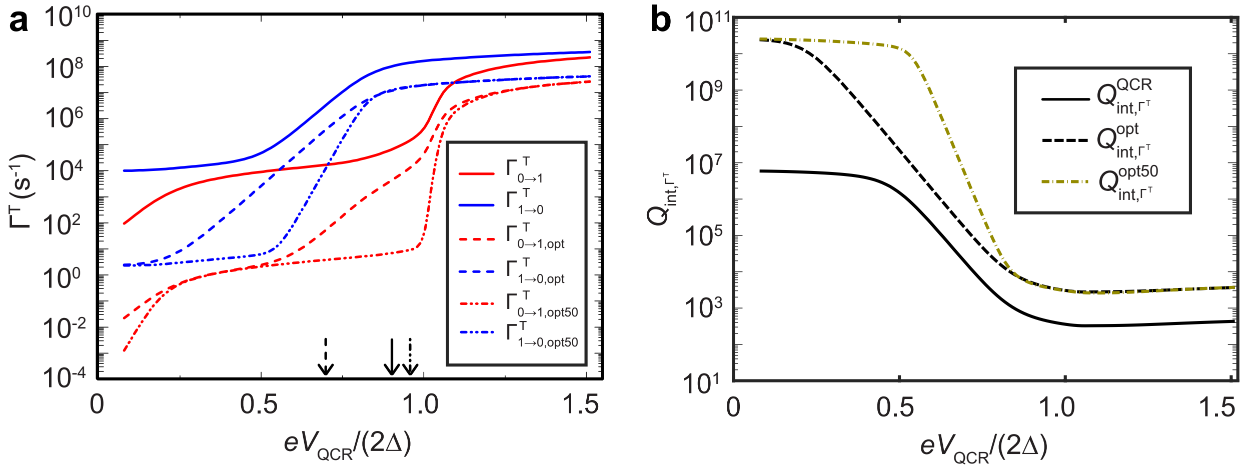

Supplementary Figure 3 | Voltage-dependent operation characteristics. **a**, Resonator excitation ($\Gamma^{\rm T}_{0\to1}$) and relaxation rates ($\Gamma^{\rm T}_{1\to0}$) as functions of the QCR operation voltage, $V_{\rm QCR}$, for the measured quantum-circuit refrigerator (QCR) sample (solid lines), the optimized sample using the measured QCR electron temperature (dashed lines), and the optimized sample using 50 mK lower electron temperatures (dash-dotted lines). See Supplementary Table 2 for the parameters of the optimized sample. Each operation voltage yielding the minimum temperature corresponding to the photon-assisted tunnelling, $T_{{\rm res},\Gamma^{\rm T}} = \hbar\omega_0/[\log(\Gamma^{\rm T}_{0\to1}/\Gamma^{\rm T}_{1\to0})k_{\rm B}]$, is denoted by an arrow. Here, the temperature assumes the value 60 mK (solid line), 50 mK (dashed line), and 31 mK (dash-dotted line). **b**, Resonator quality factor corresponding to the photon-assisted tunnelling, $Q_{{\rm int},\Gamma^{\rm T}}$, as a function of the QCR operation voltage for the three cases shown in **a**.

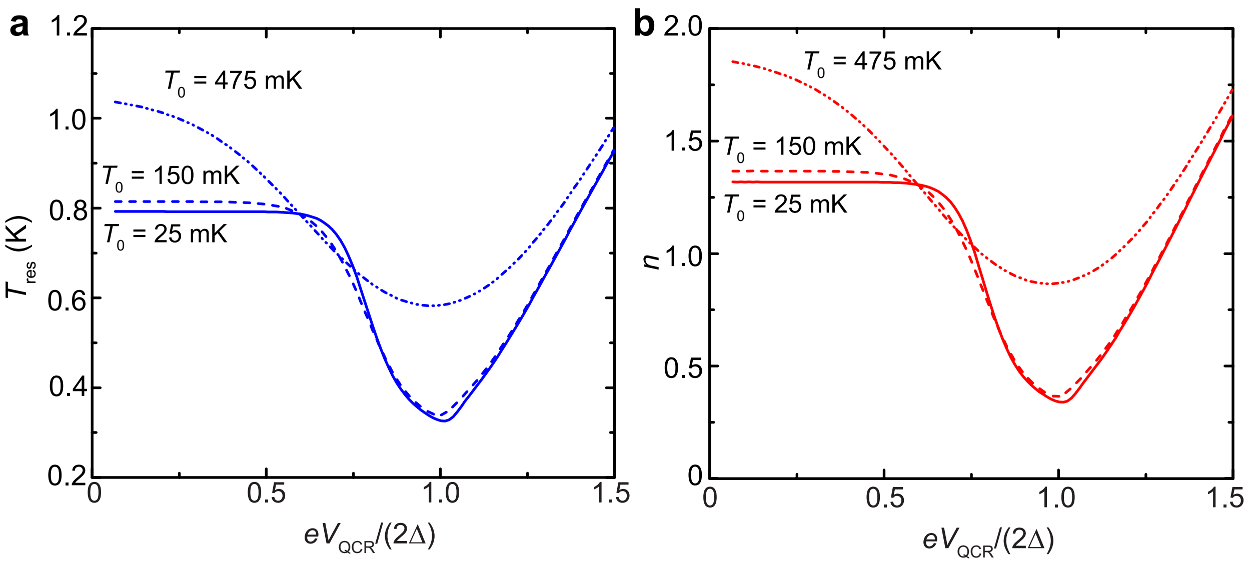

Supplementary Figure 4 | Resonator temperature and average photon number. a, Resonator temperature and **b**, average photon number as functions of the quantum-circuit refrigerator (QCR) operation voltage at phonon bath temperature of 25 mK (solid lines), 150 mK (dashed lines), and 475 mK (dash-dotted lines). The results are obtained using an upgraded thermal model where the two-state approximation for the resonator mode is not utilized. Here, we employ the experimental data of the QCR electron temperature. The simulation parameters are given in Supplementary Table 1 except for $T_x^{sat} = 64$ mK, $\Gamma_{leak} = 4.5 \times 10^7$ s^{-1}, $\Omega_{QCR} = 0.01$ μm^3, and $G_x = 1.2 \times 10^{-14}$ W/K.

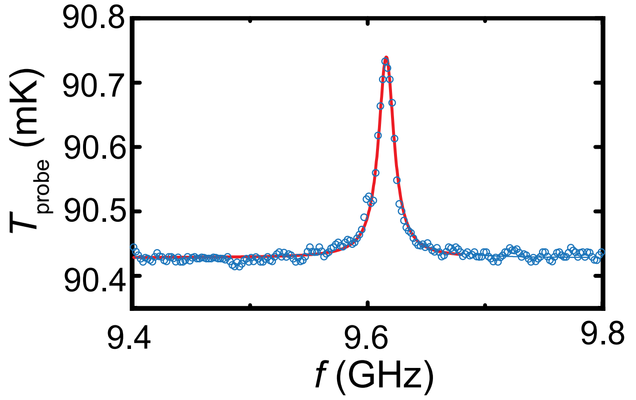

Supplementary Figure 5 | Quality factor of the control sample. Experimentally observed electron temperature at the probe resistor (markers) as a function of the frequency of the external microwave excitation. See Fig. 1a for the measurement scheme. The input power is -56 dBm at room temperature and it is attenuated according to Supplementary Fig. 1c before reaching the sample. The solid line is a Lorenzian fit to the data.

Supplementary Table 1 | **Device and model parameters.** Identical parameters are used for the active sample and for the control sample unless the specific parameter value for the control sample is given in parenthesis.

Parameter	Symbol	Value	Unit
Resonator length	L	6.833	mm
Inductance per unit length	L_l	4.7×10^{-7}	H/m
Capacitance per uni length	C_l	1.3×10^{-10}	F/m
Fundamental resonance frequency	f_0	9.32	GHz
Resistance of QCR and probe resistors	R	46	Ω
Distance of the resistors from resonator edge	x	100	μm
Volume of QCR and probe resistors	$\Omega_\text{QCR}, \Omega_\text{probe}$	$4200 \times 250 \times 20$	nm^3
Superconductor gap parameter	Δ	214 (216)	μeV
Dynes parameter	γ_D	1×10^{-4}	
Normal state junction resistance	R_T	23.4 (20.5)	kΩ
Thermometer bias current	$I_\text{th,QCR}$, $I_\text{th,probe}$	17	pA
Material parameter for Cu	Σ_Cu	2×10^9	$\text{W K}^{-5}\ \text{m}^{-3}$
Residual heating constant	α	1.5×10^{-3}	K^{-1}
Residual heating constant	β	0.38	K
Resonator constant excitation rate	Γ_leak	8.062×10^7	s^{-1}
Heat conductance to excess bath	G_x	8.8695×10^{-14}	WK^{-1}
Excess bath saturation temperature	T_x^sat	65.4 (104.5)	mK

Supplementary Table 2 | **Parameters for the optimized sample.** The resistance of the optimized quantum-circuit refrigerator (QCR) sample (see Supplementary Fig. 2) is estimated using the measured resistivity of copper in the realized sample and the revised dimensions of the copper block (3000×3000×200 nm^3). The total capacitance of the two NIS junctions in the optimized sample, $C_\mathrm{J}^\mathrm{tot}$, is obtained using a conveniently realizable junction area of 70×70 nm^2 and the usual junction capacitance per unit area 45 fF/$\mu\mathrm{m}^2$. The effective distance of the QCR from the edge of the resonator, x, is calculated from the total junction capacitance as described in Supplementary Note 4. The value of the Dynes parameter is obtained from ref. 20 for NIS junctions with proper filtering and shielding. The normal-state junction resistance can be increased compared with the realized sample by increasing the oxidation time and pressure in the fabrication process.

Parameter	Symbol	Value	Unit
Resonator length	L	6.833	mm
Inductance per unit length	L_l	4.7×10^{-7}	H/m
Capacitance per uni length	C_l	1.3×10^{-10}	F/m
Fundamental resonance frequency	f_0	9.32	GHz
Resistance of QCR resistor	R	0.3	Ω
Total junction capacitance	$C_\mathrm{J}^\mathrm{tot}$	440	aF
Distance of the resistors from resonator edge	x	3.4	μm
Volume of QCR resistor	Ω_QCR	$3000 \times 3000 \times 200$	nm^3
Superconductor gap parameter	Δ	214	μeV
Dynes parameter	γ_D	2×10^{-7}	
Normal state junction resistance	R_T	200	kΩ
Material parameter for Cu	Σ_Cu	2×10^9	W K^{-5} m^{-3}

Supplementary References

---

[1] Giazotto, F., Heikkilä, T. T., Luukanen, A., Savin, A. M. & Pekola, J. P. Opportunities for mesoscopics in thermometry and refrigeration: Physics and applications. *Rev. Mod. Phys.* **78**, 217–274 (2006).

[2] Jones, P. J., Huhtamäki, J. A. M., Tan, K. Y. & Möttönen, M. Single-photon heat conduction in electrical circuits, *Phys. Rev. B* **85**, 075413 (2012).

[3] Ingold, G.-L. & Nazarov, Y. Charge tunnelling rates in ultrasmall junctions. In Grabert, H. & Devoret, M. (eds.) *Single Charge Tunneling*, vol. 294 of *NATO ASI Series*, 21–107 (Springer US, 1992).

[4] Pekola, J. P. *et al.* Environment-assisted tunnelling as an origin of the dynes density of states. *Phys. Rev. Lett.* **105**, 026803 (2010).

[5] Giazotto, F. & Martnez-Pérez, M. J. The Josephson heat interferometer. *Nature* **492**, 401–405 (2012).

[6] Lindström, T. *et al.* Properties of high-quality coplanar waveguide resonators for QIP and detector applications. *J. Phys.: Conf. Ser.* **150**, 052140 (2009).

[7] Vissers, M. R. *et al.* Low loss superconducting titanium nitride coplanar waveguide resonators. *Appl. Phys. Lett.* **97**, 232509 (2010).

[8] Bruno, A. *et al.* Reducing intrinsic loss in superconducting resonators by surface treatment and deep etching of silicon substrates. *Appl. Phys. Lett.* **106**, 182601 (2015).

[9] Ohya, S. *et al.* Room temperature deposition of sputtered tin films for superconducting coplanar waveguide resonators. *Supercond. Sci. Technol.* **27**, 015009 (2014).

[10] Jaim, H. M. I. *et al.* Superconducting tin films sputtered over a large range of substrate dc bias. *IEEE Trans. Appl. Supercond.* **25**, 1–5 (2015).

[11] Suri, B. *et al.* Nonlinear microwave photon occupancy of a driven resonator strongly coupled to a transmon qubit. *Phys. Rev. A* **92**, 063801 (2015).

[12] Koski, J. V., Peltonen, J. T., Meschke, M. & Pekola, J. P. Laterally proximized aluminum tunnel junctions. *Appl. Phys. Lett.* **98**, 203501 (2011).

[13] Masuda, S. *et al.* Cryogenic microwave source based on nanoscale tunnel junctions. Preprint at http://arxiv.org/abs/1612.06822 (2016).

[14] Andreev, A. F. The Thermal Conductivity of the Intermediate State in Superconductors. *Sov. Phys. JETP* **19**, 1228–1231 (1964).

[15] Hekking, F. W. J. & Nazarov, Yu. V. Subgap conductivity of a superconductor˘normal-metal tunnel interface. *Phys. Rev. B* **49**, 6847–6852 (1994).

[16] Rajauria, S., Gandit, P., Fournier, T., Hekking, F. W. J. & Courtois, H. Andreev Current-Induced Dissipation in a Hybrid Superconducting Tunnel Junction. *Phys. Rev. Lett.* **100**, 207002 (2008).

[17] Greibe, T. *et al.* Are "Pinholes" the Cause of Excess Current in Superconducting Tunnel Junctions? A Study of Andreev Current in Highly Resistive Junctions. *Phys. Rev. Lett.* **106**, 097001 (2011).

[18] Averin, D. A. & Pekola, J. P. Nonadiabatic Charge Pumping in a Hybrid Single-Electron Transistor. *Phys. Rev. Lett.* **100**, 217003 (2008).

[19] Maisi, V. F, Saira, O.-P., Pashkin, Yu. A., Tsai, J. S., Averin, D. V. & Pekola, J. P. Real-Time Observation of Discrete Andreev Tunneling Events. *Phys. Rev. Lett.* **106**, 217003 (2011).

[20] Saira, O.-P., Kemppinen, A., Maisi, V. F. & Pekola, J. P. Vanishing quasiparticle density in a hybrid Al/Cu/Al single-electron transistor. *Phys. Rev. B* **85**, 012504 (2012).
